# Pathogenesis of *Candida albicans* Infections in the Alternative Chorio-Allantoic Membrane Chicken Embryo Model Resembles Systemic Murine Infections

**DOI:** 10.1371/journal.pone.0019741

**Published:** 2011-05-13

**Authors:** Ilse D. Jacobsen, Katharina Große, Angela Berndt, Bernhard Hube

**Affiliations:** 1 Department for Microbial Pathogenicity Mechanisms, Leibniz Institute for Natural Product Research and Infection Biology, Jena, Germany; 2 Institute of Molecular Pathogenesis, Friedrich-Loeffler-Institut (Federal Research Institute for Animal Health), Jena, Germany; 3 Friedrich Schiller University, Jena, Germany; Montana State University, United States of America

## Abstract

Alternative models of microbial infections are increasingly used to screen virulence determinants of pathogens. In this study, we investigated the pathogenesis of *Candida albicans* and *C. glabrata* infections in chicken embryos infected via the chorio-allantoic membrane (CAM) and analyzed the virulence of deletion mutants. The developing immune system of the host significantly influenced susceptibility: With increasing age, embryos became more resistant and mounted a more balanced immune response, characterized by lower induction of proinflammatory cytokines and increased transcription of regulatory cytokines, suggesting that immunopathology contributes to pathogenesis. While many aspects of the chicken embryo response resembled murine infections, we also observed significant differences: In contrast to systemic infections in mice, IL-10 had a beneficial effect in chicken embryos. IL-22 and IL-17A were only upregulated after the peak mortality in the chicken embryo model occurred; thus, the role of the Th17 response in this model remains unclear. Abscess formation occurs frequently in murine models, whereas the avian response was dominated by granuloma formation. Pathogenicity of the majority of 15 tested *C. albicans* deletion strains was comparable to the virulence in mouse models and reduced virulence was associated with significantly lower transcription of proinflammatory cytokines. However, fungal burden did not correlate with virulence and for few mutants like *bcr1*Δ and *tec1*Δ different outcomes in survival compared to murine infections were observed. *C. albicans* strains locked in the yeast stage disseminated significantly more often from the CAM into the embryo, supporting the hypothesis that the yeast morphology is responsible for dissemination in systemic infections. These data suggest that the pathogenesis of *C. albicans* infections in the chicken embryo model resembles systemic murine infections but also differs in some aspects. Despite its limitations, it presents a useful alternative tool to pre-screen *C. albicans* strains to select strains for subsequent testing in murine models.

## Introduction

*Candida albicans* is both a commensal on human mucosal surfaces and one of the most important human fungal pathogens [1]. Disease caused by *C. albicans* ranges from relatively benign infections of skin to oral and vaginal thrush, deep-seated mycoses and life-threatening sepsis [1]. The incidence of *Candida* sepsis has steadily risen over the last two decades and recent studies have identified *Candida* spp. as the fourth most common cause of sepsis in hospital settings [2]. Despite the availability of modern antimycotics, systemic candidiasis is still associated with high mortality rates [3].

To better understand pathogenesis and to identify fungal virulence-associated factors, complex infection models are indispensable. Murine models for both superficial and systemic *C. albicans* infections have been developed and are widely used to study pathogenesis and determine the virulence of defined *C. albicans* mutants [4], [5]. However, the use of murine models is restricted by costs, the requirement of specialized facilities and personnel, legal requirements and ethical considerations. Therefore, infection models using invertebrates, e. g. *Galleria mellonella* larvae and Toll-deficient *Drosophila melanogaster*, as alternative hosts have been developed. These alternative hosts are suitable to determine the virulence of different *C. albicans* strains and to evaluate the efficacy of antifungal compounds [6], [7], [8], [9].

Systemic infections in mice lead to a rapid induction of various cytokines, neutrophil infiltration of affected organs and finally progressive septic shock [10], [11]. This immunopathology is a major difference between murine and insect models, since insect hosts mount a defensive immune response to *C. albicans* infections based on phagocytic cells and the upregulation of antimicrobial peptides but do not develop septic shock [12], [13]. In contrast, the avian immune response to infection resembles the mammalian one to a large extend, including the production of both proinflammatory and regulatory cytokines [14] and development of a systemic response to LPS application [15].

Chicken embryos as alternative model host for fungal pathogens were already described in 1939 by Moore [16]. Until the late 1970s embryonated eggs were frequently used for research in immunology, virology, bacteriology but also mycology [17], [18], [19]. More recently, this alternative model host has also been used to investigate the virulence of *C. albicans* deletion mutants and to investigate efficacy of antimycotics [20], [21]. Infection of the chorio-allantoic membrane within embryonated chicken eggs is technically easy and thus suitable for screening purposes. However, possible pathogenesis mechanisms, the role of the host's immune system, a comparison with murine models and the potential use of this alternative host for virulence screens have not been addressed in detail. Therefore, we aimed to elucidate the pathogenesis of *C. albicans* infections and the role of the immune system in this alternative host. Furthermore, we analyzed *C. glabrata* and 15 defined *C. albicans* mutants with known virulence potential in mice with regard to virulence, fungal burden and induction of cytokines to determine whether this model is suitable for virulence screening of *C. albicans* mutants.

## Results

### Establishment and characterization of the infection model

Both preculture conditions and the host have been shown to have a significant influence on survival in systemic *C. albicans* infections in mice [22]. Previous studies using chicken embryos as alternative hosts vary in several technical aspects, thus hampering direct comparison of obtained results. Therefore, we initially thoroughly characterized the model using *C. albicans* SC5314 for infection of the chorio-allantoic membrane (CAM). Mortality was confirmed to be dose-dependent in our model in embryos infected on developmental day 10: Infection with 10^7^ cfu/egg led consistently to 75–100% mortality within 7 days. Gradual reduction of mortality was observed with log-fold dilutions of the infectious dose (data not shown). Infection with 10^5^ cfu/egg resulted in 40–60% final mortality and doses below 10^4^ cfu/egg led to no significant mortality (data not shown). Inactivation of *C. albicans* by heat-treatment or thimerosal led to survival rates similar to the PBS control (80–100%). Therefore, the LD_100_ of 10^7^ cfu/egg was used for subsequent survival comparisons. Analyses requiring a higher number of surviving embryos throughout a time course were performed using the LD_50_ of 10^5^ cfu/egg.

**Figure 1:**
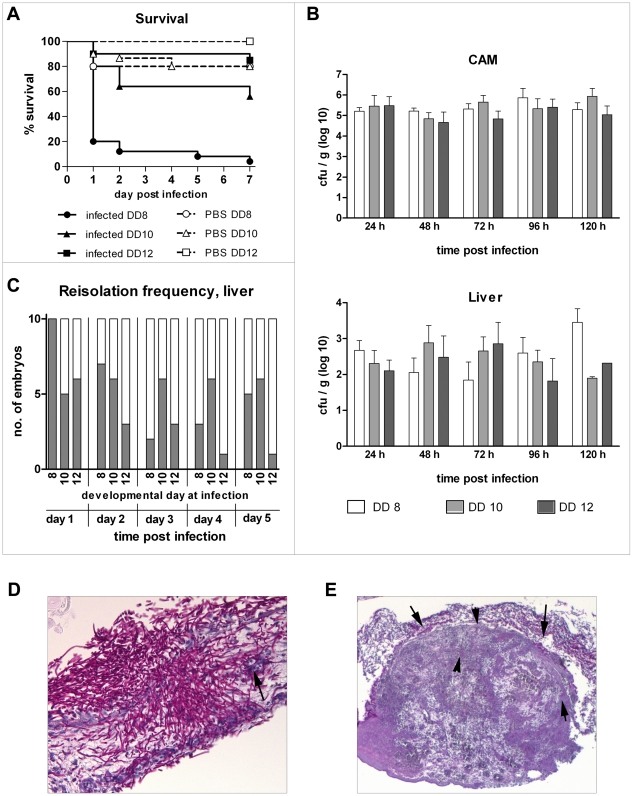
Characterization of the course of infection in chicken embryos infected with *C. albicans* SC5314 at different developmental days. (A) Mortality after infection on the CAM (Kaplan-Meyer curve). N = 20 per group per experiment, two independent experiments. Significant mortality (compared to age-matched PBS control, log rank test) was only observed for embryos infected on developmental day (DD) 8 (P<0.01). The logrank test for trend was significant for comparison of infected groups (P<0.0001). (B) Comparison of fungal burden in CAM in embryos infected at different developmental days (DD) as mean and SD, n = 10. (C) Frequency of positive isolation of *C. albicans* from liver (n = 10 per DD and time point). Dark: positve isolation; white: no fungi isolated. (D and E) Histology of embryos infected on developmental day 10. Periodic acid-Schiff stain (fungal elements: pink). (D) Hyphae invading into and penetrating the full thickness of the CAM 24 h after infection. Arrow: blood vessel penetrated by *C. albicans*. (E) Histology of macroscopically visible plaque 3 days after infection. Arrows indicate fungal cells.

The outcome of infection with various pathogens, including *C. albicans*, in chick embryos has been described to depend on the developmental stage (age) at infection [17], [23]. In agreement with these observations, we found a clear correlation of age at infection and survival. At both high (10^7^ cfu, data not shown) and low infectious doses (Fig. 1A), infection on developmental day 8 led to rapid mortality significantly higher than in embryos infected on developmental day 10 (P<0.001), while embryos infected on developmental day 12 were highly resistant and showed survival rates undistinguishable from the PBS control. It has been suggested that the increased resistance of older embryos to infection with pathogens might reflect maturation and increasing competence of the embryonic immune system [17]. Increased immune competence could enable the host to limit fungal burden and prevent dissemination of the pathogen, thus increasing survival of the host. To test this hypothesis, we determined fungal burden in the CAM and dissemination into the liver during the course of infection (day 1–5 post infection (p.i.)) in embryos infected on developmental day 8, 10 and 12 (n = 10 per day and age). *C. albicans* could be isolated from the CAM of all infected embryos (Fig. 1B). However, isolation from the liver, indicating systemic dissemination of *C. albicans*, was only sporadically successful. Although we observed a tendency towards lower dissemination rates in oldest embryos, this trend was not statistically significant (Fig. 1C). To determine whether dissemination might be an event associated with imminent death of an embryo, we additionally performed isolation from livers and complete abdomens of freshly deceased embryos. Similarly to living embryos, however, isolation was only infrequently positive (data not shown). Surprisingly, the embryonic age at infection did not have a significant impact on the number of cfu isolated from the CAM (Fig. 1B). Furthermore, cfu did not change over the course of the experiment (Fig. 1B). Neither fungi nor bacteria could be cultured from CAM and embryonic tissue of PBS-mock infected controls (n = 15), confirming the absence of contaminations.

Independent of the fungal burden and dissemination, maturation of the embryo and its organs might lead to increased resistance of tissues to damage. Therefore, we analyzed pathological alterations in embryos infected at different age macroscopically and histologically. Briefly, macroscopically visible white to grayish nodules as well as edematous areas appeared on the CAM 24–48 h p.i. Histological analysis of these alterations revealed focal, invasive, filamentous growth of *C. albicans*, often accompanied with complete destruction of the CAM and angioinvasion (Fig. 1D). In areas were hyphae had not penetrated the whole thickness of the CAM, epithelial hyperplasia, proliferation of fibroblasts and cellular infiltration of the mesoderm was observed. Infiltrates became more prominent at 72 h p.i. as yellowish plaques (Fig. 1E). The majority of examined embryos showed no pathological alterations in internal organs; necrotic lesions containing small numbers of hyphae were observed only sporadically (<10%). These findings were consistent with previous observations by others [19], [21], [24]. Importantly, quality and quantity of the observed alterations were comparable between embryos infected at different developmental stages.

### Embryonic maturation influences cytokine production after *C. albicans* infection and susceptibility to endotoxins

**Figure 2:**
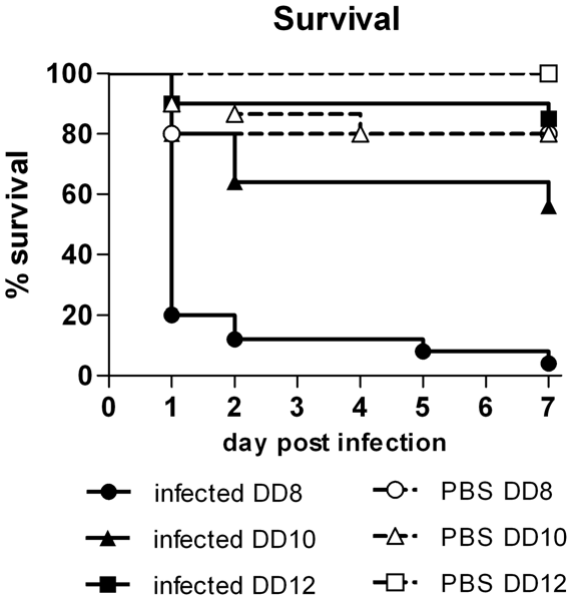
Age-dependent mortality after application of 100 **µ**g LPS on the CAM. Survival is shown as Kaplan-Meyer curve, n = 20 per group per experiment, two independent experiments. Significant mortality (compared to age-matched PBS control, log rank test) was only observed for embryos infected on developmental day (DD) 8 (P<0.001) and DD10 (P<0.01). The log rank test for trend was significant for comparison of LPS groups (P<0.005).

In mice, systemic infection with *C. albicans* leads to progressive sepsis [10]. Using *E. coli* LPS applied onto the CAM, we confirmed previous observations by Smith and Thomas [25] that chicken embryos are susceptible to lethal septic shock and that this susceptibility is age-dependent (Fig. 2). Therefore, we hypothesized that an age-dependent sepsis-like response might contribute to the age-dependency of mortality in embryos infected with *C. albicans*. To test this hypothesis, we determined the transcription levels of cytokines, which have been implicated in pathogenesis in mice [11], [26], in the CAM of embryos challenged either with LPS or *C. albicans*. Cytokines function as mediators for immune cell recruitment, activation and immune modulation during infection and are involved in the development of sepsis [27].

**Figure 3:**
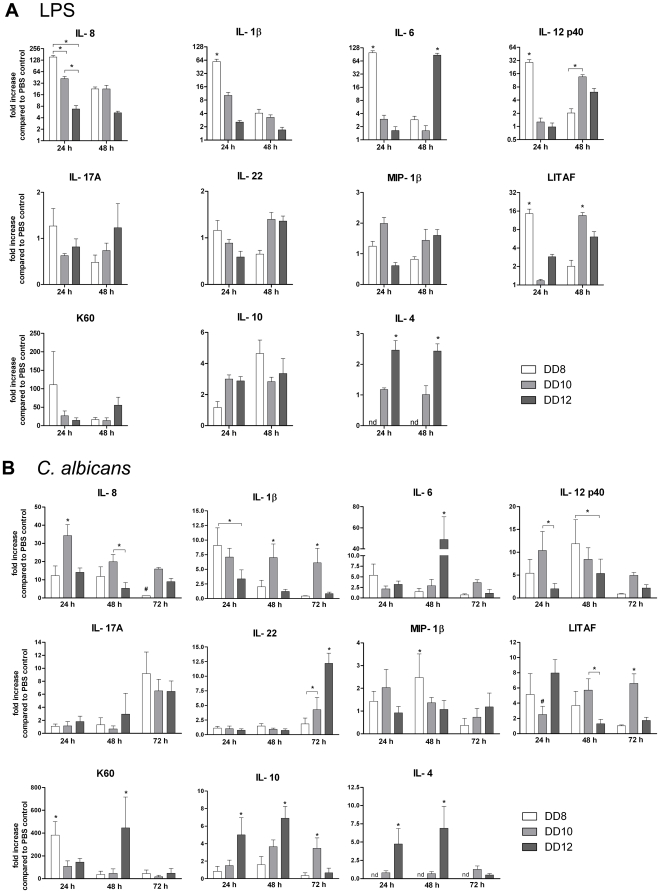
Influence of embryonic age on the transcriptional levels of cytokines after LPS application and infection with *C. albicans* SC5314. Embryos were either challenged with 100 µg LPS (A) or infected with 10^5^ cfu *C. albicans* on the CAM (B). N = 5 per challenge and time point, data is shown as mean and SD. DD: developmental day. Asterisks indicate statistically significant differences (P<0.05; 2-way ANOVA and Bonferroni post test).

Because mortality after LPS application occurred predominantly within the first 24 h and no deaths were observed after 48 h (Fig. 2), the expression of cytokine genes was analyzed 24 h and 48 h p.i. (Fig. 3A). Upregulation of the proinflammatory cytokines IL-8 (attracting mainly monocytes in chickens), IL-1β, IL-6, IL-12β p40 (note that the subunit p40 is shared with IL-23), the TNFα homologue LITAF (LPS-induced tumor necrosis factor) and K60 (functional orthologue of murine KC; K60 attracts mainly heterophils, the avian homologue of neutrophils) was significantly higher 24 h p.i. in embryos challenged on developmental day 8 compared to older embryos. Similarly, these cytokines (with the exception of LITAF) were upregulated to a greater extend in embryos challenged on day 10 compared to day 12. However, the difference between these two age groups was only significant for IL-8. The kinetics of proinflammatory cytokine gene transcription also differed depending on age: In youngest embryos, transcription levels were consistently highest 24 h p.i. In contrast, transcription of IL-12β p40 and LITAF increased from 24 h to 48 h in embryos challenged on day 10 or 12 and transcription of K60 in oldest embryos was highest 48 h after challenge. IL-6 was only moderately induced at both time points in embryos challenged on day 10 whereas older embryos showed a strong upregulation after 48 h. Age-dependent differences were also observed for IL-10 and IL-4, which exhibit regulatory rather than proinflammatory functions: IL-10 was already upregulated after 24 h in older embryos while the youngest embryos showed upregulation only after 48 h. IL-4 transcripts were upregulated in embryos challenged on developmental day 12 whereas embryos challenged on developmental day 10 transcribed only basal levels of IL-4. In the youngest embryos IL-4 transcripts could not be detected. IL-17A, IL-22 and MIP-1β were not significantly upregulated in any embryo (Fig. 3A). Transcription of IFN-γ was unaltered in all groups at all time points. IL-2 transcripts could not be detected in any group (data not shown). Application of zymosan (consisting of fungal glucan) likewise resulted in age-dependent survival and similar cytokine transcription patterns. However, large doses of zymosan (1 mg/egg) were needed to induce mortality and the transcription level of proinflammatory cytokines was lower than with LPS (data not shown). While fungal glucan can stimulate a proinflammatory response, we cannot exclude that low-level contamination with endotoxins contributed to the observed effects.

Similarly, the cytokine transcription profiles upon infection with *C. albicans* differed depending on the age of embryos at infection (Fig. 3B). In contrast to LPS, however, only IL-1β and K60 showed highest transcription in youngest embryos 24 h p.i. IL-1β transcription levels declined in embryos infected on developmental day 8 and 12 from 24 h to 48 h whereas embryos infected on developmental day 10 showed continuous high transcription levels throughout 24 h to 72 h p.i. K60 displayed different induction kinetics in oldest embryos where strongest transcription was observed 48 h p.i. IL-8 was most strongly transcribed in embryos infected on day 10. The age groups did not differ in IL-6 induction 24 h p.i., but IL-6 transcription 48 h p.i. was significantly increased in the oldest embryos. Differences to LPS challenge were also observed for IL-12β p40 transcription: Strongest transcription in embryos infected on day 8 was only seen after 48 h, while embryos infected on day 10 responded more rapidly. IL-12β p40 transcription in embryos infected on day 12 was overall lower than in the other age groups. LITAF transcription increased over time in embryos infected on day 10 whereas older embryos showed highest induction 24 h p.i. Comparable to challenge with LPS, transcription of IFN-γ was unaltered in all groups and IL-2 transcripts were not detectable (data not shown). MIP-1β was only moderately (maximum two-fold) upregulated without significant differences between age groups. Transcription of IL-17A and IL-22 increased only 72 h p.i. While this upregulation was comparable amongst age groups for IL-17A, IL-22 transcripts were significantly more abundant in older embryos. Transcription of the regulatory cytokine IL-10 24 h and 48 h p.i. was significantly higher in embryos infected on day 12 compared to day 10. Consistent with the results of LPS challenge, IL-4 transcription only increased in embryos infected on day 12 and transcripts were not detectable in embryos infected on day 8.

This data demonstrate that the age of the host at challenge or infection has a significant influence on the transcriptional cytokine response. The proinflammatory response to LPS declines with increasing age. The response to *C. albicans* infection shows some similarities to LPS challenges, but also displays different kinetics which partially depend on the developmental stage of the embryo. In both LPS challenge and infection with *C. albicans*, increasing age led to stronger transcription of regulatory cytokines.

### Modulation of inflammation by IL-10 enhances survival of embryos after infection

**Figure 4:**
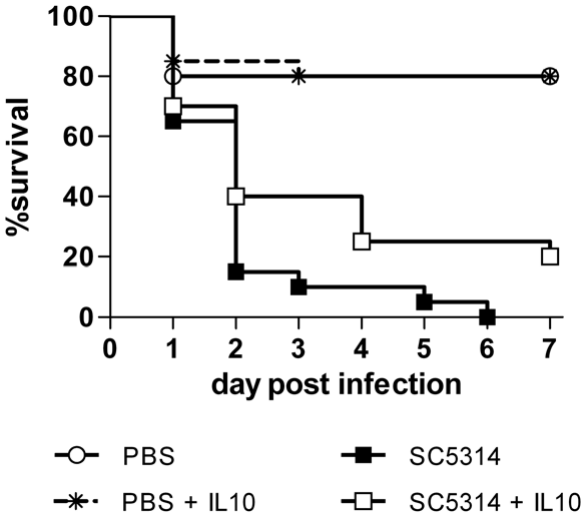
Addition of IL10 reduces mortality after *C. albicans* infection. On developmental day 10, embryos were treated with 100 ng recombinant chicken IL10 (IL10) in PBS/BSA or PBS/BSA alone ( ) and infected with 10^7^ cfu *C. albicans* SC5314. Survival is shown as Kaplan-Meyer curve, n = 20 per group, two independent experiments. Treatment had a significant effect on survival (P<0.05, log rank test).

Age-dependent resistance of chicken embryos challenged with LPS or infected with *C. albicans* correlated with the transcription levels of IL-10 and IL-4. In mice, the role of IL-10 is not fully clear. While MacCallum et al. showed an inverse correlation of IL-10 with kidney lesion severity and suggested that IL-10 might exert a defensive role [11], Vazquez-Torres et al. demonstrated increased susceptibility of IL-10 knock out mice to systemic candidiasis [28]. To clarify the role of IL-10 in infected chicken embryos, we applied 100 ng recombinant chicken IL-10 onto the CAM of 10 days old eggs 15 min prior to infection. Controls were treated with carrier solution only. IL-10 application alone had no influence on survival of non-infected control embryos. In infected embryos, IL-10 treatment had a minor but reproducible and significant positive effect on survival (P<0.05) (Fig. 4).

### *Candida glabrata* survives and disseminates in infected embryos but does not cause mortality

In contrast to *C. albicans*, systemic *C. glabrata* infections in mice do not lead to mortality although the fungus is able to persist within the host [29]. To determine whether chicken embryos as alternative hosts are comparable to mice in this respect, we analyzed the outcome of infection with *C. glabrata* in the egg model.

**Figure 5:**
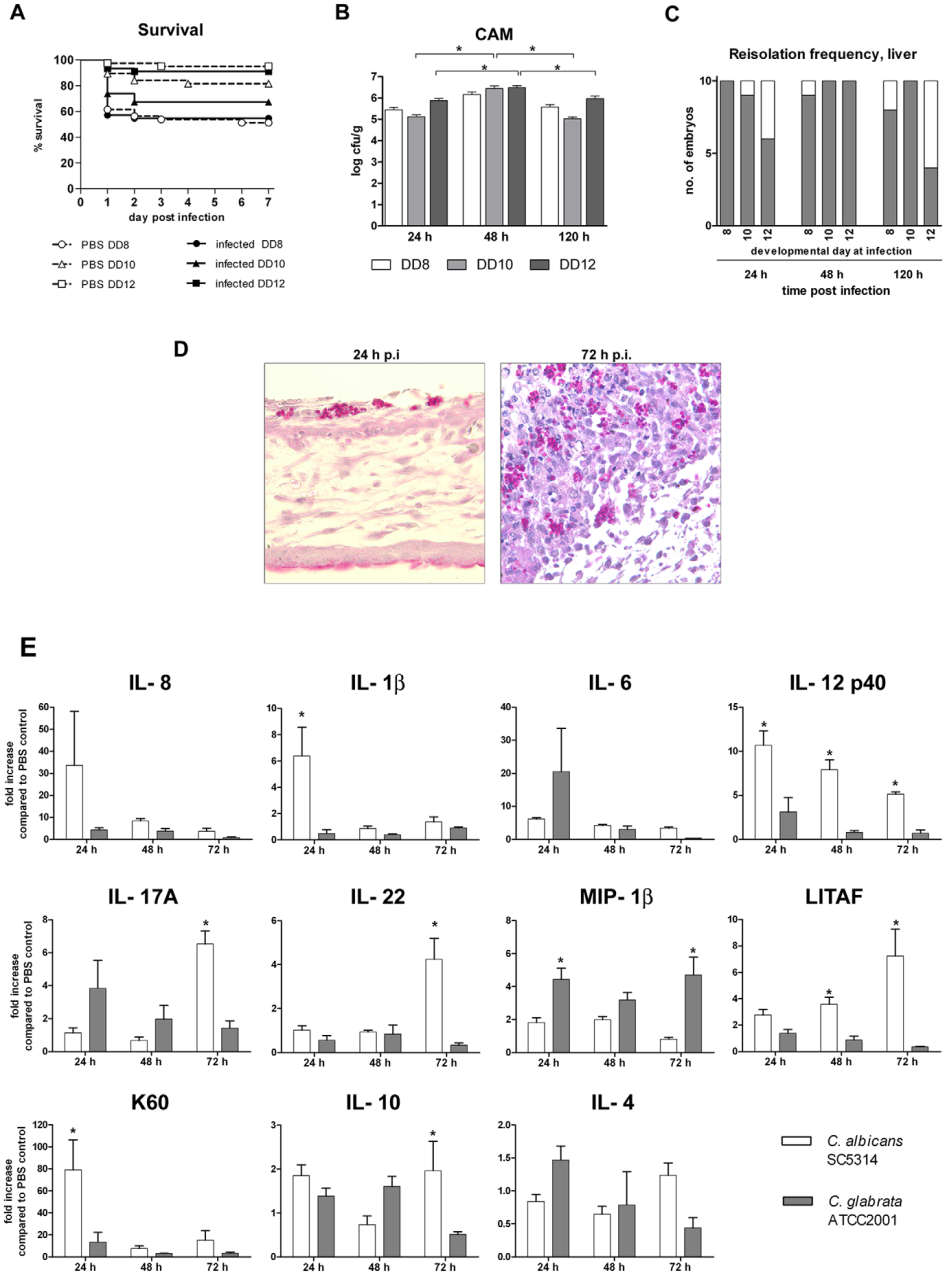
Characterization of the course of infection in chicken embryos infected with *C. glabrata* ATCC2001. (A) Mortality after infection on the CAM (Kaplan-Meyer curve). N = 20 per group per experiment, two independent experiments. No significant mortality (compared to age-matched PBS control, log rank test) was observed, independent of the developmental day (DD) at infection. (B) Comparison of fungal burden in CAM, n = 10 per group, mean and SD. (C) Frequency of positive isolation in livers of embryos infected at different DD as mean and SD. Dark: positve isolation; white: no fungi isolated. (D) Representative histology of *C. glabrata* infected CAM. (E) Comparison of cytokine transcription in embryos infected with either *C. albicans* SC5314 (white) or *C. glabrata* ATCC2001 (grey) on DD10. N = 5 per time point, data is shown as mean and SD. Asterisks indicate statistically significant differences (P<0.05; 2-way ANOVA and Bonferroni post test).

Independent of the developmental stage at infection, *C. glabrata* infected embryos showed survival almost identical to PBS mock-infected controls (Fig. 5A). However, *C. glabrata* was able to survive and replicate in association with the CAM (Fig. 5B) without significant differences between the age groups. Dissemination occurred frequently in all age groups (Fig. 5C) and *C. glabrata* was isolated from 90–100% of livers 48 h p.i. Histologically, *C. glabrata* was found on and within the CAM (Fig. 5D). Plaque formation occurred earliest 72 h p.i. These plaques were characterized by cellular infiltrations into the mesoderm (Fig. 5D). Thus, although lacking hyphae-dependent penetration mechanisms, *C. glabrata* was able to enter deeper tissues and, similar to mice, *C. glabrata* persisted within infected embryos without gross pathological alterations and mortality.

In contrast to *C. albicans*, *C. glabrata* infections in mice induce only a transient increase in proinflammatory cytokines and very moderate influx of immune cells [29], [30]. To determine whether similar differences in the host response to *C. albicans* and *C. glabrata* occur in infected chicken embryos, we compared transcription levels of various cytokines and chemokines after infection with either *C. albicans* or *C. glabrata* in embryos infected on developmental day 10.

The expression levels of IL-8, IL-1β, IL12 p40 and K60 in *C. glabrata* infected embryos 24 p.i. were only moderately upregulated and lower than in *C. albicans* infected embryos (Fig. 5E). LITAF transcripts were moderately induced both in *C. albicans* and *C. glabrata* infected embryos 24 h p.i. However, while LITAF transcription decreased over time to PBS control levels in *C. glabrata* infected embryos, an increase was observed after infection with *C. albicans* (Fig. 5E). Surprisingly, IL-6, IL-17A and MIP-1β transcripts were higher in *C. glabrata* infected embryos 24 h p.i. (Fig. 5E). However, IL-17A decreased over time in embryos infected with *C. glabrata* while an increase was observed in embryos infected with *C. albicans*. IL-2 transcripts were not detectable in either group. IFN-γ transcription levels in both groups were comparable to PBS controls. IL-10 was upregulated in both groups without quantitative difference (data not shown). Thus, comparable to murine hosts and epithelial models, *C. glabrata* induces overall less proinflammatory cytokines than *C. albicans*.

### Embryonated eggs as alternative hosts to determine virulence of *C. albicans* gene deletion mutants

One of the most commonly used applications of alternative model hosts is virulence screening of strains or mutants to select strains of interest for subsequent infection experiments using mammalian hosts. It was shown that chicken embryos are generally suitable for virulence determination of different *Candida* species and *C. albicans* gene deletion mutants [18], [21]. However, only few *C. albicans* mutants with known virulence potential in mice were tested. To gain more comprehensive information on the suitability of the model as a screening tool and possible mechanisms which influence virulence of strains in the chicken embryo model, we analyzed 15 known *C. albicans* gene deletion mutants for their ability to kill chicken embryos. The mutants were selected because of described defects in damaging epithelial cells and/or attenuation in murine infection models. Virulence of the two relevant parental strains, CAI4+pCIp10 and BWP17+pCIp30, was comparable to SC5314 in the embryo model (analyzed by Kaplan-Meyer curves and log rank test, data not shown). Additionally, for mutants, which showed significant attenuation, the complemented mutant, as far as available, was tested.

In survival comparison over a 7 day observation period, ten out of 15 mutants tested showed significantly (P<0.05) attenuated virulence in the chicken embryo model (Table S1). All tested complemented strains led to lower survival rates than infection with the corresponding homozygous deletion mutant, but 3 out of 9 tested complemented strains were significantly reduced in virulence in comparison to the parental strain (Table S1). Notably, kinetics of mortality onset differed between the mutants: Within the first 24 h, most mutants (*mnt1*Δ, *sap1–3*Δ, *rim101*Δ, *dfg16*Δ, *als3*Δ, *bcr1*Δ, *eed1*Δ) caused similar mortality as the respective parental strain but very few or no deaths in the following days. In contrast, only few embryos died within 24 h after infection with *efg1*Δ and *efg1*Δ*cph1*Δ. Within the first 24 h, *tpk2*Δ caused mortality comparable to the wild type, followed by a delay in killing. However, final mortality rates at the end of the observation period were comparable between *tpk2*Δ and the parental strain CAI4+pCIp10, emphasizing the necessity of a sufficiently long observation period.

**Figure 6:**
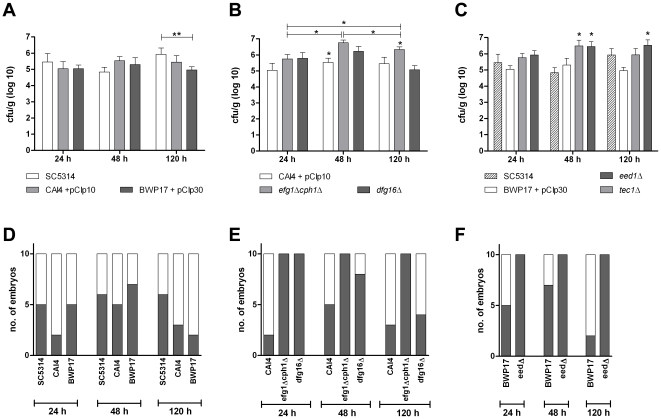
Fungal burden and dissemination frequencies of *C. albicans* mutants in infected embryos. Embryos were infected on developmental day 10 with 10^5^ cfu *C. albicans* SC5314. (A to C) Fungal burden in the CAM (n = 10 per strain and time point). (D to F) Dissemination frequencies based on positive isolation from the liver (n = 10 per strain and time point). Dark: positive isolation; white: no fungi isolated. (A, D) Comparison of SC5314, CAI4+pCIP10 and BWP17. (B, E) CAI4-derived mutants with altered fungal burden and/or dissemination frequencies. (C, F) BWP-derived mutants with altered fungal burden and/or dissemination frequencies.

To determine whether reduced virulence of mutants was associated with reduced fungal survival in infected eggs, fungal burden was determined as colony forming units (cfu) in the CAM and dissemination into the liver was analyzed 24 h, 48 h and 120 h after infection with *als3*Δ, *bcr1*Δ, *dfg16*Δ, *eed1*Δ, *efg1*Δ*cph1*Δ, *mnt1*Δ, *rim101*Δ, *tec1*Δ, *sap1–3*Δ and the parental strains CAI4+pCIP10 (CAI4) and BWP17+pCIp30 (BWP17). Similar cfu of SC5314 and the parental strains CAI4 and BWP17 could be isolated from the CAM 24 h and 48 h p.i. However, the burden of BWP17 was significantly lower than of SC5314 120 h p.i (Fig. 6A). Dissemination of these strains occurred infrequently without significant differences between the strains (Fig. 6D).

All mutants could be readily isolated from the CAM and no mutant showed reduced fungal burden within the CAM. In comparison to their respective parental strains, significantly higher cfu of *efg1*Δ*cph1*Δ, *dfg16*Δ, *tec1*Δ and *eed1*Δ were recovered from the CAM of infected eggs (Fig. 6B and C). Dissemination frequencies were similar for most mutants and their parental strain except for *efg1*Δ*cph1*Δ, *eed1*Δ and *dfg16*Δ. *efg1*Δ*cph1*Δ and *eed1*Δ could be isolated from all cultured livers, while dissemination of *dfg16*Δ occurred more frequently within the first 24 h p.i. only (Fig. 6E and F). In summary, these results suggested that (i) increased clearance is unlikely to be the cause of reduced virulence, (ii) dissemination does not necessarily lead to embryonic death and (iii) reduced ability to form hyphae increases likelihood of dissemination.

**Figure 7:**
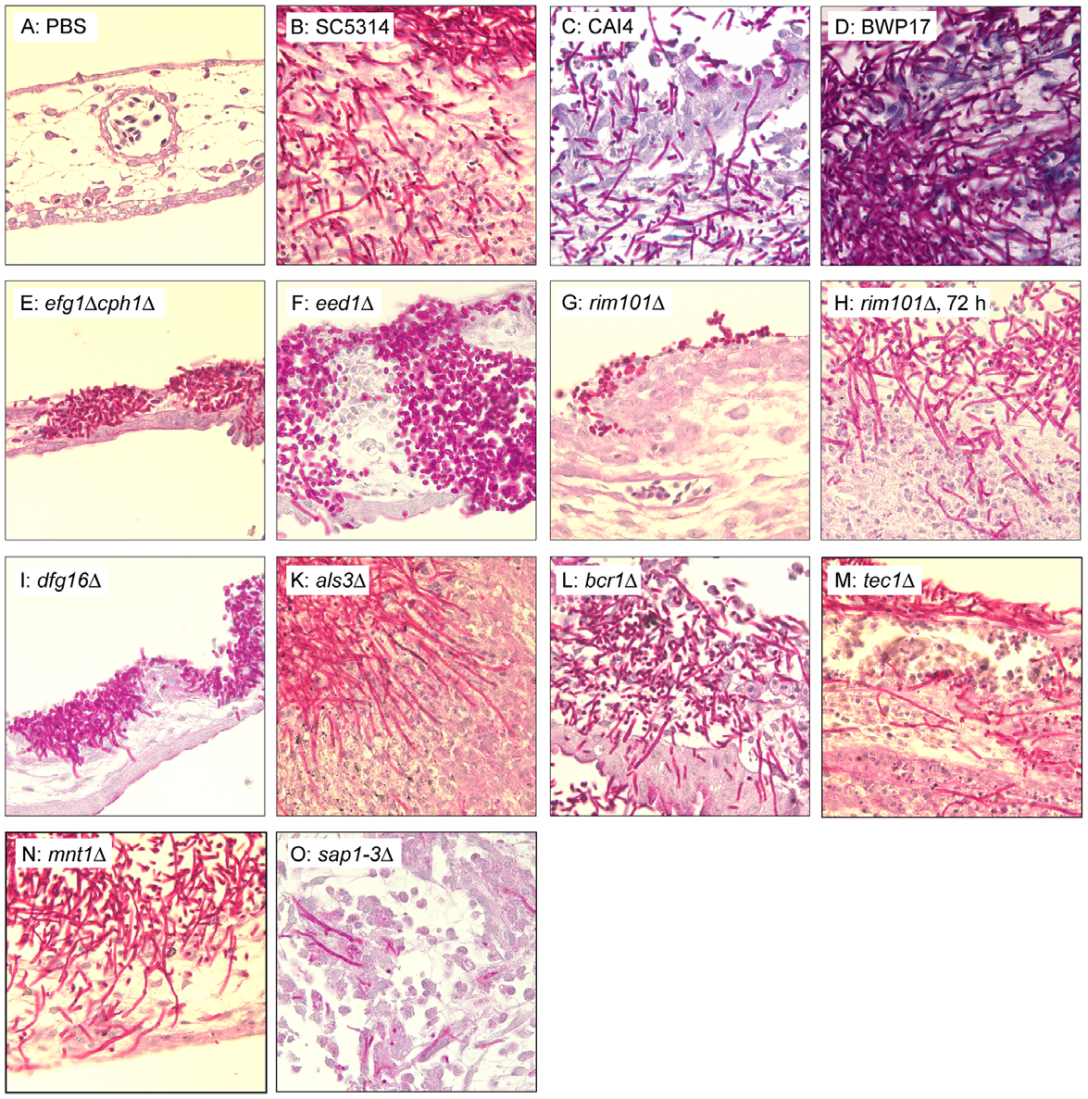
Fungal morphology during infection of the CAM. Histological sections stained with PAS 24 h (except H: 72 h) after infection with 10^5^ cfu. 63× magnification.

Hyphae formation is an important virulence factor of *C. albicans* [31]. Consistently, mutants with defects in hyphae formation (*efg1*Δ*cph1*Δ, *eed1*Δ, *dfg16*Δ, *rim101*Δ, *ras1*Δ) were attenuated in the chicken embryo model (Table S1). However, filament formation depends on various stimuli and defects in hyphae formation can be overcome by appropriate stimuli. This has been shown for *tec1*Δ, which is deficient in hyphae formation *in vitro* but forms wild type-like filaments *in vivo* in the murine kidney after intravenous infection [32]. To confirm known morphology defects of mutants, we analyzed the fungal morphology in the CAM by histology 24 h, 48 h, and 72 h p.i. As described previously [21], wild type and parental strains formed hyphae invading into the CAM within the first 24 h (Fig. 7B–D). Yeast cells were rarely observed in these strains. In contrast, *efg1*Δ*cph1*Δ grew as yeast only (Fig. 7E). Only few, short filaments were observed in *eed1*Δ (Fig. 7F). Interestingly, despite the obvious defect in hyphae formation and similar to *C. glabrata*, both strains were regularly located within the mesoderm of the CAM, supporting that a hyphae-independent invasion mechanism exists in this model. In contrast, *rim101*Δ was found predominantly as yeast cells on the surface of the CAM (Fig. 7G) and invasion was observed only infrequently 72 h p.i. in association with sparse hyphae development (Fig. 7H). Consistent with *in vitro* results [33], *dfg16*Δ produced only short hyphae and showed reduced invasion into the CAM (Fig. 7I). Both *als3*Δ and *bcr1*Δ were able to produce filaments and invade the CAM (Fig. 7K and L). However, compared to the parental strains, more yeast cells were present in these strains and invasion occurred less frequently. *tec1*Δ, *sap1–3*Δ and *mnt1*Δ all formed filaments but showed differences in frequency and depth of invasion: While *mnt1*Δ and *tec1*Δ (Fig. 7M and N) frequently penetrated the full width of the CAM, *sap1–3*Δ was predominantly found on the surface of the CAM and invading filaments were only occasionally and in few numbers observed within the mesoderm (Fig. 7O).

**Figure 8:**
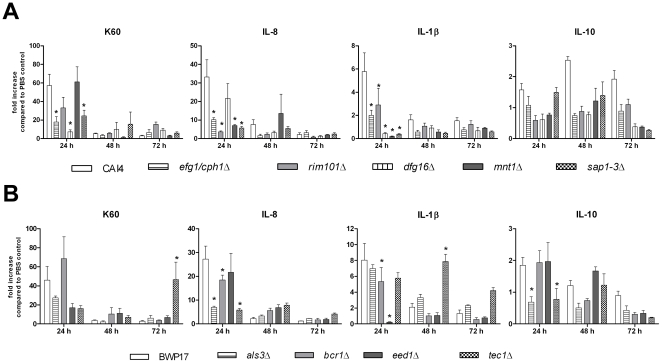
Cytokine transcription in the CAM after infection with *C. albicans* mutants. N = 5 per time point, data is shown as mean and SD. Asterisks indicate statistically significant differences (P<0.05; 2-way ANOVA and Bonferroni post test). (A) CAI4-derived mutants with altered cytokine transcription profiles. (B) BWP-derived mutants with altered cytokine transcription profiles.

In intravenously challenged mice, induction of proinflammatory cytokines in kidneys is directly correlated with the virulence of *C. albicans* strains [11], [26]. To assess whether a similar correlation exists in chicken embryo infection, we determined K60, IL-8, IL-1β and IL-10 transcription in the CAM of embryos infected with *C. albicans* deletion mutants in comparison to their parental strain. Attenuated *C. albicans* mutants generally induced less proinflammatory cytokine transcripts 24 p.i. (Fig. 8), but specific differences were observed between mutants: Only *efg1*Δ*cph1*Δ and *sap1–3*Δ showed reduced transcription levels of all three proinflammatory cytokines (Fig. 8A). *mnt1*Δ and *rim101*Δ induced significantly less IL-8 and IL-1β but levels of K60 were not or only moderately reduced, respectively (Fig. 8A). A similar induction pattern was observed for *bcr1*Δ (Fig. 8B). *dfg16*Δ and *eed1*Δ induced high levels of IL-8 but reduced levels of K60 and IL-1β (Fig. 8A and B). Wildtype-like IL-1β induction but less K60 and IL-8 transcripts were observed with *als3*Δ. Finally, *tec1*Δ differed in transcription kinetics from both the parental strain and the other mutants by showing a reduced induction 24 h p.i. but a strong late induction of IL-1β (48 h p.i., Fig. 8B) and K60 (72 h p.i., Fig. 8B). IL-10 was only moderately induced by CAI4 and BWP and most mutants showed no increase compared to the PBS control (Fig. 8).

## Discussion

Mammalian infection models, in particular mouse models, are commonly considered the gold standard to study host-pathogen interactions of human pathogens. However, the use of mammalian models is limited by several factors, including ethical considerations, costs and requirement of specialized facilities. Under these conditions, alternative model hosts of a lower phylogenetic or ontogenetic stage, like invertebrates or avian embryos, can provide an important and alternative tool to study virulence. Chicken embryos have been used previously to investigate *C. albicans* virulence and morphological alterations during infection have been described [20], [21], [23], [24]. Nonetheless, it is of yet unclear which alterations lead to mortality, how the host responds and to which extend interaction of *C. albicans* with the embryo reflects the well studied murine models.

It should be noted that we did not screen buffers and chemicals used in our study for potential endotoxin contamination. Therefore, we cannot completely exclude that endotoxin contaminations might have influenced results obtained in this study. However, all buffers used were tested in a control group of chicken embryos (PBS control) in each individual experiment. The survival of PBS controls was comparable with sham operated embryos (data not shown). The low mortality observed in PBS controls thus appears to be attributable to the necessary mechanical manipulations. All cytokine data were calculated as fold increase to the above mentioned PBS controls within the same experiment to obviate buffer-mediated changes in cytokine transcription.

We aimed at characterizing the host-pathogen interaction during *C. albicans* infection in embryonated eggs to elucidate the potential use of this model as a screening tool for virulence of *C. albicans*. We confirmed previous observations by others that *C. albicans* readily colonizes and invades the CAM, including blood vessels. While Gow et al. [21] could not detect dissemination of SC5314 into the embryo, we observed infrequent dissemination. Given the small size of the liver in young embryos and the low fungal burdens, methodological differences can easily influence detection of dissemination and might therefore explain the differing results. Using a different wild type *C. albicans* strain, Fox et al. [23] observed an age-dependent decrease in fungal burden in the liver, whereas we observed a trend but no significant age-dependency. Our observation that *C. glabrata* and some hyphae-deficient *C. albicans* mutants (*efg1*Δ*cph1*Δ, *eed1*Δ, *dfg16*Δ) disseminate at high frequency suggests that strain specific differences in dissemination exist and supports the hypothesis that yeast cells are the morphological form responsible for dissemination. Additionally, our results confirm the conclusion of Gow et al. [21] that dissemination is no necessary prerequisite for lethal outcome after infection of the CAM.

While dissemination frequencies and the fungal burden in the liver and CAM per weight did not change significantly with increasing age, we observed a strong decrease in mortality in older embryos. Thus, age-dependent susceptibility is unlikely to be an effect of changes in the ratio of infectious dose to embryonic body weight. Likewise, the fungal burden per weight in the CAM did not change depending on embryonic age and during the course of infection, suggesting that (i) at an early stage, the embryonic immune system is able to control fungal proliferation but incapable of clearing infection and (ii) that other mechanisms than clearance of fungi mediate resistance.

In systemically infected mice, the local proinflammatory cytokine response in the kidneys correlates with kidney lesions and lethality [11], [26]. Similarly, we observed higher proinflammatory cytokine production in younger, more susceptible chicken embryos, suggesting that immunopathology in the chicken embryo model contributes to pathogenesis. This hypothesis is further supported by our finding that *C. glabrata*, which does not cause significant mortality in infected chicken embryos, and attenuated *C. albicans* mutants induce significantly less proinflammatory cytokines than *C. albicans* wild type strains, consistent with reports from systemic murine candidiasis [11], [29]. Moreover, the age-dependent susceptibility to LPS-induced septic shock in chicken embryos mirrors susceptibility to *C. albicans* infection. Although a Th2 response is generally considered detrimental in systemic candidiasis, IL-4 deficiency in mice enhances susceptibility to systemic murine *C. albicans* infection [28]. Thus, the age-dependent ability of chicken embryos to increase IL-4 transcription upon infection might contribute to age-dependent resistance. However, whether IL-4 directly influences susceptibility in chicken embryos or if the embryo's ability to produce IL-4 is only an indicator of a more complex response involving other effectors, remains to be determined. In contrast to the role of IL-10 in mice, where deletion of IL-10 is protective against systemic candidiasis [28], we observed a protective effect of addition of IL-10 to chicken embryos at a susceptible age. Since the general function of IL-10 is modulation and downregulation of proinflammatory responses, the protective effect of IL-10 in chicken embryos could be mediated by an immune-modulatory function which downregulates an otherwise destructive proinflammatory response, thus differing from its role in candidiasis in mice. We suggest that the key to protection is the appropriate balance of the immune response: The proinflammatory response must be sufficient to control the fungus but inflammation needs to be restricted to avoid excessive inflammation-mediated damage.

In addition to Th1, a Th17 response is essential for a protective immune response against disseminated candidiasis in mice [34]. While one study showed increased IL-17A levels in mice 24 h p.i. [34], IL-17 could not be detected within the first 48 h in another study [11]. In chicken embryos, we only observed transcriptional upregulation of IL-17A at 72 h p.i. Similarly, IL-22, another cytokine which has been implicated in Th17 responses, was only upregulated after 72 h. Thus, even though chicken embryos mount a measurable Th17 response on the transcriptional level, it occurs only after main mortality occurred. Furthermore, only IL-22 showed transcriptional differences depending on embryonic age at the time of infection. Therefore, in contrast to mice, IL-17A and IL-22 may not directly contribute to the reduced susceptibility of older chicken embryos.

Macroscopically, *C. albicans* infections lead to the formation of plaques on the CAM. These plaques are granuloma-like structures [24], which are typical for an avian immune response. In contrast to neutrophil infiltration in mammals, which often leads to the formation of abscesses and tissue destruction, heterophil infiltration in birds is resolved by demarcation of necrotic heterophils (and pathogens) by epithelioid macrophages and fibroblasts. The resulting granuloma-like structure isolates pathogens and potential harmful heterophil components from the surrounding tissue [35]. Formation of granuloma-like structures in the *C. albicans* chicken embryo model is likely triggered by the early increase in chemoattractant cytokines, especially IL-8 and K60, which recruit macrophages and heterophils to the site of infection. Visible plaque formation coincides with the decline in cytokine transcription, suggesting that successful demarcation of the pathogen removes the immune stimulus. Moreover, the majority of deaths in the chicken embryo model occur before mature granuloma are formed. Thus, we suggest that while a proinflammatory response in the CAM is necessary to demarcate infected areas, an imbalanced, excessive cytokine response contributes to deaths of young embryos after *C. albicans* infection. In older embryos, the mounted immune response appears to be more balanced and sufficiently induces granuloma formation without causing additional harm.

As cytokines function as signal molecules in infection, the defensive efficacy of the immune response is mediated by immune cells, antibacterial peptides and the complement system. Although we did not analyze effector functions in this study, others have shown that antibacterial peptides are differentially expressed during ontogenesis and that the phagocytic capacity increases during embryonic development [23], [36]. Therefore, it appears likely that functional maturation of immune effector mechanisms additionally contributes to increasing resistance. Furthermore, we cannot exclude that younger embryos are more sensitive to damage of the CAM. Although the quality and quantity of lesions within the CAM was comparable between embryos of different age, higher sensibility of younger embryos could additionally contribute to the influence of embryonic age on the outcome of infection.

To gain further insights into the host-pathogen interaction and to determine the value of chicken embryos as alternative hosts for virulence determination, we analyzed several *C. albicans* deletion mutants for their ability to kill chicken embryos, invade the CAM and induce proinflammatory cytokine transcription. Interestingly, all mutants were isolated in similar or higher cfu compared to the parental strain, suggesting that observed virulence defects are due to altered pathogenesis rather than decreased fitness. In accordance with the role of hyphae and hyphae-associated gene expression in other infection models [31], hyphae-deficient mutants were attenuated in the chicken embryo model. Furthermore, these mutants induced less proinflammatory cytokine transcripts in the chicken embryo model, consistent with the observations of Moyes et al., that hyphae formation and invasion are necessary to trigger cytokine production in cell culture models [37].

Different murine models mimicking different manifestations of human candidiasis are used to investigate the role of putative virulence factors, but only few *C. albicans* mutants have been tested in several murine models. For *TPK2* and *CKA2*, the role in virulence appears to depend on the murine infection model used, as the respective deletion mutants are fully virulent in murine systemic candidiasis, but attenuated in oropharyngeal candidiasis and epithelial cell models [38], [39], [40]. In chicken embryos, *tpk2*Δ led to delayed mortality, but showed no significant attenuation and *cka2*Δ was fully virulent. Thus, virulence of these two mutants in chicken embryos grossly resembled systemic but not mucosal infection in mice. In contrast, *tec1*Δ is attenuated in the systemic mouse model [32], but fully virulent in the chicken embryo model. It has been suggested that the attenuation in the systemic mouse model is due to the decreased ability to evade macrophages after phagocytosis [32]. However, in contrast to intravenous infection which directly exposes the fungus to phagocytic cells, there are no residential macrophages within the CAM, thus delaying the exposure of fungal cells to phagocytes until these cells immigrate from the blood. Thus, a disadvantage of the CAM infection model is that it might not appropriately mimic initial survival in the blood stream after systemic infection. While intravenous infection of chicken embryos is possible [24], it is technically more challenging than infection of the CAM and therefore not suitable for screening purposes. Interestingly, *tec1*Δ induced a delayed increase in proinflammatory cytokines compared to the parental strain. The cause for this delay is not clear, but since Tec1 acts as a transcriptional regulator, deletion of *TEC1* might influence the stimulation of host cells via altered expression of surface-associated molecules. For example, Tec1 indirectly influences transcriptional activation of *ALS3* via Bcr1 [41].

The transcription factor Bcr1 is important for biofilm formation *in vitro* and *in vivo* [42]. Part of these biofilm defects are mediated by Bcr1-dependent regulation of Als3, a well studied adhesin, invasin and ferritin receptor [42], [43], [44]. Despite its role for biofilm formation *in vivo*, *bcr1*Δ is not essential for virulence in a systemic mouse model [42]. In contrast, we observed significant attenuation of both *bcr1*Δ and *als3*Δ in the chicken embryo model. These differences could be explained by the different inoculation routes: Application onto the CAM requires adhesion of fungal cells to the CAM and the typical growth of *C. albicans* in foci on the CAM suggests that aggregation of fungal cells is involved in establishment of infection. Biofilm-related mechanisms might be involved in this step. Furthermore, reduced induction of proinflammatory cytokines by both *bcr1*Δ and *als3*Δ might also contribute to attenuation.

Glycosylation of secreted and surface proteins influences filamentation, adhesion, cell wall stability, and interaction with host cells [45], [46]. In contrast to Gow et al. [21], we found the o-mannosyltransferase mutant *mnt1*Δ to be attenuated in chicken embryos. These discrepancies might be due to differences in methodology, like the age of embryos at infection, since glycosylation is involved in the interaction of *C. albicans* with macrophages [45] and the ability of embryonic macrophages to kill *C. albicans* increases with age [23]. Furthermore, attenuation of *mnt1*Δ only became evident after a prolonged observation period while mortality rates after 24 h were indistinguishable between *mnt1*Δ and its parental strain. Thus, rating mortality over several days is necessary to determine the virulence potential of strains.

Conflicting results have also been published regarding the role of secreted aspartic proteases (Saps) for virulence of *C. albicans* in chicken embryos. Saps are expressed during infection of chicken embryos [21] and protease-deficient *C. albicans* strains were found to be attenuated in virulence and unable to invade the CAM [47]. Furthermore, Kobayashi et al. [48] described that a purified *Candida* protease disrupts intercellular junctions of the CAM and protease-treatment as well as damage to the CAM allowed a protease-deficient *C. albicans* strain to invade. However, differences in virulence were found amongst protease-producing strains [47] and Gow et al. [21] found *SAP* deletion mutants to be fully virulent in chicken embryos. In our hands, a *sap1–3*Δ mutant was significantly attenuated while a *sap4–6*Δ mutant was fully virulent. Histology revealed normal hyphae formation of *sap1–3*Δ but reduced numbers of hyphae invading into deeper layers of the CAM and reduced induction of proinflammatory cytokines. While methodological differences might account for some of the variation we also cannot exclude polar effects in the *sap1–3*Δ mutant due to the use of the *URA*-blaster method in mutant generation. However, it also appears plausible that proteases might aid *C. albicans* in initial invasion into the CAM but that other factors determine the final outcome of infection.

Complex genetic effects might likewise contribute to the observation that some of the complemented mutants tested showed only incomplete recovery of the wild type virulence phenotype. Commonly, only a single copy of the deleted gene is reintroduced into a mutant. Thus, gene dosage effects might influence virulence of a complemented strain. The locus at which the wild type gene is reintroduced and unspecific effects due to more rounds of genetic manipulation performed to construct complemented strains might also affect virulence in this model. While incomplete rescue of virulence has also been described for some strains in murine models [49], it appears to be more common in the chicken embryos model. This should be taken into consideration when evaluating results obtained with the chicken embryo model.

*C. glabrata* lacks virulence in moderately immunosuppressed mice [29] and chicken embryos although it's ability to cause lethal infections in humans is well documented [2]. Therefore, both murine models and chicken embryos as alternative hosts are obviously not ideal systems to study pathogenesis of human candidiasis caused by *C. glabrata*.

While it is apparent that we do not fully understand pathogenesis in chicken embryos infected with *C. albicans* on the CAM yet, based on our data and previous work published by others, we propose the following model: The initial phase after application of *C. albicans* is determined by the fungal ability to adhere and form microcolonies on the CAM. In the second phase, rapid hyphae formation and invasion into the CAM triggers a strong proinflammatory host response. An imbalanced, sepsis-like immune response in combination with immature effector functions of the embryonic immune system and damage of the CAM putatively contribute to mortality. Invasion of blood vessels is frequently observed in this stage but dissemination into internal organs of the embryo occurs only infrequently and does not contribute significantly to pathogenesis. If the embryo survives the second phase, foci of fungal invasion are demarcated by immune cells and fibroblasts, visible as plaques, thus limiting further expansion of invasive fungal growth without eliminating the pathogen. Therefore, fungal burden remains stable over time and does not correlate with survival. This demarcation phase coincides with a decline of the proinflammatory response and decrease of mortality frequency in surviving embryos.

In summary, infection of the CAM combines aspects of invasion assays and murine systemic infection in a complex *in vivo* model using an alternative vertebrate host. The advantages of the CAM model lie in comparatively low costs and the technical simplicity with no need for specialized facilities and expertise. The main differences between the chicken embryo model and systemic murine models lie in the application route and the immune status of the host. Systemic infections in mice are predominantly performed in immunocompetent animals, whereas chicken embryos should be considered naturally immunocompromised. Although the immune response of chicken embryos and mice are similar with regard to the induction of proinflammatory cytokines, the effect of IL-10 clearly differs in the two models. Furthermore, the role of IL17A, and a Th17 response in general, remain unclear in the chicken embryo model. In addition, candidiasis in mice generally leads to abscess formation while chicken embryos demarcate infected areas by granuloma. This difference might explain why attenuation of strains in the chicken embryo model was not accompanied by reduction of fungal burden. As the age of chicken embryos at infection has a major influence on the outcome, preincubation and infection procedures need to be standardized to gain comparable results. Our results indicate that attenuation of mutants might be less pronounced in the embryo model compared to systemic murine infections and that complementation might not sufficiently rescue virulence phenotypes in all cases. Thus, the chicken model might yield false-negative results. However, the model still provides a suitable screening tool to determine the virulence of large numbers of mutants strains to identify attenuated strains for subsequent testing in murine models. Furthermore, if specialized animal facilities are not available, the chicken embryo model can be employed as alternative *in vivo* system. In combination with survival analysis, histology and determination of the cytokine response, the CAM chicken model provides insights into pathogenesis. If applied to testing *C. albicans* mutants, this information aids in formulating hypotheses for subsequent refined testing in appropriate mammalian models.

## Materials and Methods

### Ethics statement

All experiments were performed in compliance with the German animal protection law. According to this, no specific approval is needed for work performed in avian embryos before the time of hatching. Experiments were terminated latest on developmental day 18, three days before hatching, by chilling the eggs on ice for 30–60 min.

### *Candida* strains and preparation for infection

The *Candida* strains used in this study have been described before and are listed in Table S2. For infection experiments, strains were subcultured once on YPD agar plates for 24 h at 37°C. A single colony was then inoculated into 20 ml liquid YPD and cultured for 12–14 h at 30°C with 200 rpm. Cells were harvested by centrifugation (3,000× g, 5 min, 4°C) and washed twice with cold sterile phosphate buffered saline (PBS), pH 7.4. After determining the cell number with a Cellometer™ Auto T4 (Nexcelom Bioscience, Lawrence, MA, U.S.A.), the suspensions were adjusted to the desired concentration with cold, sterile PBS. Inocula were maintained on ice and used within 1–2 h. The cell numbers in inocula were confirmed by plating serial dilutions on YPD agar plates. Colony forming units (cfu) were determined after 36 h incubation at 30°C. Experiments were only considered valid if the difference between calculated cell number and counted cfu was <20%.

### Inactivation of *C. albicans*

For heat inactivation, *C. albicans* suspension adjusted to the appropriate concentration were incubated for 15 min at 80°C. Alternatively, diluted fungal suspensions were inactivated by adding 100 mM thimerosal (Sigma, Steinheim, Germany) and incubating for 45 min at 37°C. Following thimerosal treatment, fungal cells were washed 3× with sterile PBS. Inactivation was confirmed by plating serial dilutions on YPD agar plates.

### Preparation of embryonated eggs and inoculation on the chorio-allantoic membrane (CAM)

Fertilized chicken eggs of the breed “weiße Leghorn” were obtained from a local producer. The eggs were stored at 8°C for a maximum of seven days prior to incubation at 37.6°C and 50–60% relative humidity in a specialized incubator (BSS 300, Grumbach, Germany). Eggs were turned four times a day starting on the fourth day of incubation until infection. Prior to infection, vitality of the embryo was assessed by candling. For survival experiments and determination of fungal burden, inoculation on the CAM was performed as described previously [50]. Twenty eggs were infected per group and survival was monitored for up to 7 days by candling. Experiments were repeated at least two times. Survival data were plotted as Kaplan-Meyer curves and statistically analyzed by log rank test using Graph Pad Prism Version 5.00 for Windows (GraphPad Software, San Diego California USA).

For determination of the immune response and histological analyses, the technique was modified to allow easier localizing of the site of infection: 50 µl *Candida* inoculum containing 1×10^5^ cells was placed onto sterile nylon membrane filters (0.45 µm pore size, 13 mm in diameter, cut in half; Carl Roth GmbH, Karlsruhe, Germany) on sterile Whatman® blotting paper (GE Healthcare, München, Germany). After excessive liquid had soaked through the membranes, the wet membranes were placed onto YPD agar plates until inoculation. Embryonated eggs were prepared as described above but the hole at the longitudinal side was elongated to a 5 mm slit. The inoculated membrane was carefully inserted through the slit with the inoculated site facing the CAM. Survival curves obtained using the membrane method were comparable to survival after inoculation of suspensions containing the same number of fungal cells.

Age-matched PBS mock-infected embryos were used as negative controls in all experiments.

### Application of lipopolysaccharides, zymosan and IL-10

Lipopolysaccharides (LPS, prepared by phenol extraction from *Escherichia coli* O55:B5, Sigma) and zymosan (prepared from *Saccharomyces cerevisiae*, Sigma) were solved in sterile PBS. Chicken recombinant IL-10 (Kingfisher Biotech Inc., St. Paul, MN, U.S.A.) was solved in sterile PBS containing 0.2% BSA (Serva, Heidelberg, Germany) as carrier protein. Both LPS, zymosan and IL-10 were applied onto the CAM as described above for survival experiments.

### Determination of fungal burden

Embryonated eggs were infected with 10^5^ cfu/egg as described above for survival experiments. At given time points, viable embryos (defined by embryonic movement at candling) were humanely sacrificed by chilling the eggs on ice for 30–60 min. The eggs' surface was then disinfected with 70% ethanol. The shell was cut in half with sterile scissors. Approximately 0.5–1 g CAM (comprising 1/3 to 1/2 of total CAM), were removed. The embryo was then carefully removed from its surrounding membranes and placed in 70% ethanol for 2–3 min. Using a sterile set of scissors and forceps, the abdominal cavity was opened to remove the liver. Samples were homogenized in 2 ml sterile, cold PBS using an Ultra-Turrax® T10 basic (IKA® GmbH, Staufenberg, Germany). Undiluted homogenate (liver: 4×500 µl; CAM: 100 µl) and serial dilutions were plated onto YPD agar plates and incubated for 36 h prior to colony counting. Fungal burden of CAM and liver were determined for at least ten embryos per fungal strain and time point. Statistical analysis was performed by 2-way ANOVA and Bonferroni post test using GraphPad Prism® Version 5.00 for Windows. Dissemination frequencies were compared by chi-square test.

### Histological analysis

For histological analysis, embryonated eggs were infected with the nylon membrane method and dissected as described above 24 h, 48 h and 72 h post infection (p.i.). The CAM underneath and beside the membrane was removed *in toto* with the nylon membrane, placed into biopsy bags and fixed in neutral formalin (Histofix®, Roth). Paraffin-embedded specimens were cut into 5 µm sections, stained with periodic acid-Schiff (PAS) stain according to standard protocols and examined using bright field microscopy at the indicated magnification.

### Determination of cytokine and chemokine transcription levels

For analysis of cytokine and chemokine transcription levels, embryonated eggs were infected with *C. albicans* by the nylon membrane method and dissected as described above at the indicated time points. Samples were taken and processed as previously described [50]. Quantitative real time RT-PCR reactions were set up with the One step RT qPCR MasterMix Plus for SYBR® GreenI kit (Eurogentec, Seraing, Belgium) according to the manufacturer's instructions using 100 ng total RNA as template. Primers and temperature protocols used have been published previously [50], [51], [52] with the exception of IL-10. For IL-10 the following primers were used with an annealing temperature of 56°C: forward 5′- AGGAGACGTTCGAGAAGATGGAT -3′; reverse 5′- TCACTTCCTCCTCCTCATCAGC-3′. The amplification and detection of specific products was performed on a 7300 Real Time PCR System (Applied Biosystems, Foster City, USA) using the dissociation curve mode to check the specificity of amplification products. Specific mRNA levels were quantified using the threshold method [53] and normalized to GAPDH (ΔC_T_ values). The results are expressed as n-fold change to age-matched PBS control embryos by calculating 2^(−ΔΔCT)^. The groups at a given time point were compared by 1-way ANOVA followed by Bonferroni post test (for three groups) or unpaired, two-tailed *t* test (for two groups) using Graph Pad Prism Version 5.00 for Windows.

## Supporting Information

Table S1 Virulence of C. albicans strains in embryonated eggs.
(DOC)

Table S2 Fungal strains used in this study.
(DOC)
