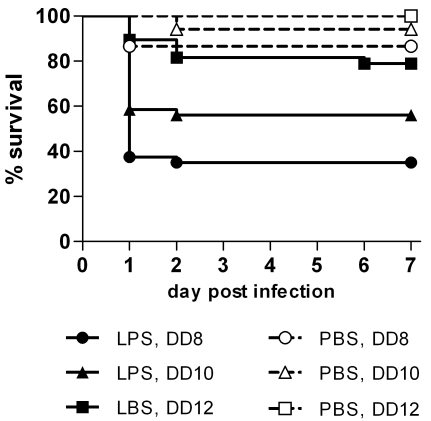# Correction: Pathogenesis of *Candida albicans* Infections in the Alternative Chorio-Allantoic Membrane Chicken Embryo Model Resembles Systemic Murine Infections

**DOI:** 10.1371/annotation/1912450a-b5a7-4dbe-bc2a-cd93579fb0dd

**Published:** 2011-06-07

**Authors:** Ilse D. Jacobsen, Katharina Große, Angela Berndt, Bernhard Hube

Figure 1 and 2 are incorrect versions. The correct Figure 1 can be viewed here: 

**Figure:**
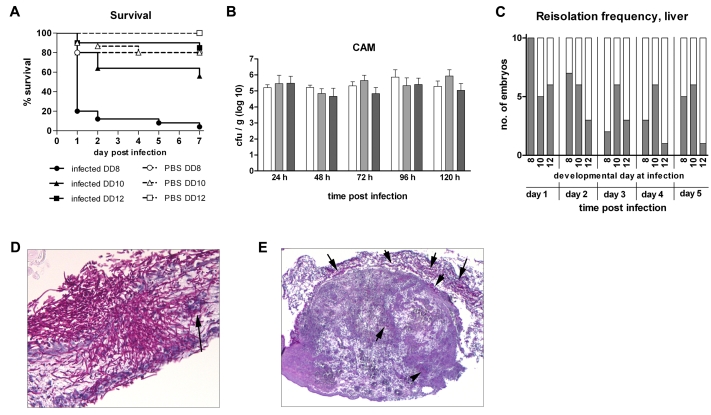


. The correct Figure 2 can be viewed here: 

**Figure:**