# Supplementary material for: Pathogenesis of Candida albicans Infections in the Alternative Chorio-Allantoic Membrane Chicken Embryo Model Resembles Systemic Murine Infections
Source: PLoS One. 2011 May 13;6(5):e19741. doi: 10.1371/journal.pone.0019741 (PMC3094387; doi:10.1371/journal.pone.0019741)
Supplement: Table S1 — Virulence of C. albicans strains in embryonated eggs. (DOC) [file pone.0019741.s001.doc]

**TABLE S1.** Virulence of *C. albicans* strains in embryonated eggs.

| **Function** | **Strain** | **% survival mutant -**  **% survival parental strain** | | **Median survival time^a^** | | **Virulence phenotype** | |
| --- | --- | --- | --- | --- | --- | --- | --- |
|  |  | **48 h p. i.** | **7 days p. i.** | **mutant** | **Parental^b^** | **In ovo** | **Mouse model** |
| morphology | *eed1*Δ | + 37.5% | + 35.0% | undefined | 2.0 | significant attenuation | strongly attenuated in intraperitoneal model (unpublished data) |
|  |  |  |  |  |  | *P* < 0.001 to wt |  |
|  |  |  |  |  |  | not sign. different to complemented strain |  |
|  | *eed1*Δ::EED1 | + 20.0% | + 20.0% | 3.0 | 2.0 | no significant attenuation | fully virulent in intraperitoneal model (unpublished data) |
|  | *efg1*Δ | + 44.6% | + 48.0% | undefined | 1.0 | significant attenuation | attenuated in systemic model [1] and intraperitoneal model [2] |
|  |  |  |  |  |  | *P* < 0.001 to wt |  |
|  |  |  |  |  |  | *P* < 0.005 to complemented strain |  |
|  | *efg1*Δ::EFG1 | + 11.3% | + 17.0% | 2.0 | 1.0 | significant attenuation | less virulent than wt in systemic model but more virulent than *efg1*Δ [1] |
|  |  |  |  |  |  | *P* < 0.05 to wt |  |
|  | *efg1*Δ*cph1*Δ | + 42.9% | + 44.9% | undefined | 1.0 | significant attenuation | strongly attenuated in systemic model [3] and attenuated in intraperitoneal model [2] |
|  |  |  |  |  |  | *P* < 0.001 to wt |  |
|  | *cph1*Δ | + 11.3% | + 10.0% | 1.0 | 1.0 | no significant attenuation | fully virulent in systemic model [1] and intraperitoneal model [2] |
|  | *ras1*Δ | + 27.0% | + 34.6% | 4.0 | 2.0 | significant attenuation | attenuated in systemic model [4]^c^ |
|  |  |  |  |  |  | *P* < 0.001 to wt |  |
|  |  |  |  |  |  | not sign. different to complemented strain |  |
|  | *ras1*Δ::RAS1 ^c^ | + 22.0% | + 23.0% | 3.5 | 2.0 | significant attenuation | fully virulent in systemic model [4]^d^ |
|  |  |  |  |  |  | *P* < 0.05 to wt |  |
|  | *tec1*Δ | + 7.5% | - 7.5% | 3.0 | 2.0 | fully virulent | attenuated in systemic model [5] and intraperitoneal model [6] |
|  | *tpk2*Δ | + 25.0% | + 10.0% | 2.0 | 1.0 | no significant attenuation | attenuated in oropharyngeal model [7] but no clear attenuation in systemic model [7,8] |
| cell surface | *mnt1*Δ | + 30.0% | + 41.6% | undefined | 2.0 | significant attenuation | reduced virulence in systemic model but not significantly attenuated [9] |
|  |  |  |  |  |  | *P* < 0.001 to wt |  |
|  |  |  |  |  |  | *P* < 0.005 to complemented strain |  |
|  | *mnt1*Δ::MNT1 | + 22.5% | + 10.0% | 2.0 | 2.0 | no significant attenuation | fully virulent in systemic model [9] |
| pH sensing and regulation | *rim101*Δ | + 34.0% | + 47.0% | undefined | 2.0 | significant attenuation | attenuated in systemic model [10] |
|  |  |  |  |  |  | *P* < 0.001 to wt |  |
|  |  |  |  |  |  | not sign. different to complemented strain |  |
|  | *dfg16*Δ | + 33.3% | + 40.0% | 3.5 | 2.0 | significant attenuation | attenuated in systemic model [11] |
|  |  |  |  |  |  | *P* < 0.001 to wt |  |
|  |  |  |  |  |  | not sign. different to complemented strain |  |
|  | *dfg16*Δ::DFG16 | + 8.3% | + 18.3% | 1.0 | 2.0 | no significant attenuation | no significant attenuation in systemic model [11] |
| proteases | *sap1-3*Δ | + 35.0% | + 41.3% | undefined | 2.0 | significant attenuation | attenuated in systemic model [12] but fully virulent in intraperitoneal model [13] |
|  |  |  |  |  |  | *P* < 0.001 to wt |  |
|  | *sap4-6*Δ | - 7.5% | - 5.0% | 1.0 | 2.0 | fully virulent | attenuated in in systemic model [14] and intraperitoneal model [13] |
| adhesion and biofilm | *als3*Δ | + 25.0% | + 30.0% | undefined | 2.0 | significant attenuation | no *in vivo* virulence data published |
|  |  |  |  |  |  | *P* < 0.01 to wt |  |
|  |  |  |  |  |  | *P* < 0.05 to complemented strain |  |
|  | *als3*Δ::ALS3 | + 7.5% | + 2.5% | 3.5 | 2.0 | fully virulent | no *in vivo* virulence data published |
|  | *bcr1*Δ | + 16.7% | + 28.3% | undefined | 2.0 | significant attenuation | reduced biofilm formation *in vivo* but fully virulent in systemic mouse model [15] |
|  |  |  |  |  |  | *P* < 0.01 to wt and complemented strain |  |
|  | *bcr1*Δ::BCR1 | + 2.0% | - 0.5% | 2.0 | 2.0 | fully virulent | normal biofilm formation *in vivo*; no *in vivo* virulence data published [15] |
| kinase | *cka2*Δ ^a^ | + 7.5% | + 7.5% | 2.0 | 1.5 | fully virulent | fully virulent in systemic model but attenuated in an oropharyngeal model [16] |

^a^ median survival time in days

^b^ for parental strain: background in Table 1; median survival time determined from experiments directly comparing mutant and parental strain, the same data was used to determine significance of virulence differences by log rank test

^c^ 2 experiments

^d^ different strains

**References:**

1. Lo HJ, Kohler JR, DiDomenico B, Loebenberg D, Cacciapuoti A, et al. (1997) Nonfilamentous *C. albicans* mutants are avirulent. Cell 90: 939-949.

2. Felk A, Kretschmar M, Albrecht A, Schaller M, Beinhauer S, et al. (2002) *Candida albicans* hyphal formation and the expression of the Efg1-regulated proteinases Sap4 to Sap6 are required for the invasion of parenchymal organs. Infect Immun 70: 3689-3700.

3. Wilson RB, Davis D, Mitchell AP (1999) Rapid hypothesis testing with *Candida albicans* through gene disruption with short homology regions. J Bacteriol 181: 1868-1874.

4. Leberer E, Harcus D, Dignard D, Johnson L, Ushinsky S, et al. (2001) Ras links cellular morphogenesis to virulence by regulation of the MAP kinase and cAMP signalling pathways in the pathogenic fungus *Candida albicans*. Mol Microbiol 42: 673-687.

5. Schweizer A, Rupp S, Taylor BN, Rollinghoff M, Schroppel K (2000) The TEA/ATTS transcription factor CaTec1p regulates hyphal development and virulence in *Candida albicans*. Mol Microbiol 38: 435-445.

6. Staib P, Binder A, Kretschmar M, Nichterlein T, Schroppel K, et al. (2004) Tec1p-independent activation of a hypha-associated *Candida albicans* virulence gene during infection. Infect Immun 72: 2386-2389.

7. Park H, Myers CL, Sheppard DC, Phan QT, Sanchez AA, et al. (2005) Role of the fungal Ras-protein kinase A pathway in governing epithelial cell interactions during oropharyngeal candidiasis. Cell Microbiol 7: 499-510.

8. Sonneborn A, Bockmuhl DP, Gerads M, Kurpanek K, Sanglard D, et al. (2000) Protein kinase A encoded by TPK2 regulates dimorphism of *Candida albicans*. Mol Microbiol 35: 386-396.

9. Munro CA, Bates S, Buurman ET, Hughes HB, Maccallum DM, et al. (2005) Mnt1p and Mnt2p of *Candida albicans* are partially redundant alpha-1,2-mannosyltransferases that participate in O-linked mannosylation and are required for adhesion and virulence. J Biol Chem 280: 1051-1060.

10. Davis D, Edwards JE, Jr., Mitchell AP, Ibrahim AS (2000) *Candida albicans* RIM101 pH response pathway is required for host-pathogen interactions. Infect Immun 68: 5953-5959.

11. Thewes S, Kretschmar M, Park H, Schaller M, Filler SG, et al. (2007) In vivo and ex vivo comparative transcriptional profiling of invasive and non-invasive *Candida albicans* isolates identifies genes associated with tissue invasion. Mol Microbiol 63: 1606-1628.

12. Hube B, Sanglard D, Odds FC, Hess D, Monod M, et al. (1997) Disruption of each of the secreted aspartyl proteinase genes SAP1, SAP2, and SAP3 of *Candida albicans* attenuates virulence. Infect Immun 65: 3529-3538.

13. Kretschmar M, Hube B, Bertsch T, Sanglard D, Merker R, et al. (1999) Germ tubes and proteinase activity contribute to virulence of *Candida albicans* in murine peritonitis. Infect Immun 67: 6637-6642.

14. Sanglard D, Hube B, Monod M, Odds FC, Gow NA (1997) A triple deletion of the secreted aspartyl proteinase genes SAP4, SAP5, and SAP6 of *Candida albicans* causes attenuated virulence. Infect Immun 65: 3539-3546.

15. Nobile CJ, Andes DR, Nett JE, Smith FJ, Yue F, et al. (2006) Critical role of Bcr1-dependent adhesins in *C. albicans* biofilm formation in vitro and in vivo. PLoS Pathog 2: e63.

16. Chiang LY, Sheppard DC, Bruno VM, Mitchell AP, Edwards JE, Jr., et al. (2007) *Candida albicans* protein kinase CK2 governs virulence during oropharyngeal candidiasis. Cell Microbiol 9: 233-245.
