# Supplementary material for: Pathogenesis of Candida albicans Infections in the Alternative Chorio-Allantoic Membrane Chicken Embryo Model Resembles Systemic Murine Infections
Source: PLoS One. 2011 May 13;6(5):e19741. doi: 10.1371/journal.pone.0019741 (PMC3094387; doi:10.1371/journal.pone.0019741)
Supplement: Table S2 — Fungal strains used in this study. (DOC) [file pone.0019741.s002.doc]

**TABLE S2.** Fungal strains used in this study.

| **Strain** | **Background** | **Genotype or description^a^** | **Source or reference** |
| --- | --- | --- | --- |
| ATCC2001 |  | *C. glabrata* wild type strain | ATCC; received from Ken Haynes |
| SC5314 |  | *C. albicans* wild type strain | [1] |
| CAI4 + pCIp10 |  | Δ*ura3:: λimm434 / URA3:: Δura3:: λimm434 RPS10 /* Δ*rps10*::pCIP10 | [2,3] |
| BWP17 + pCIp30 |  | Δ*ura3:: λimm434 / URA3:: Δura3:: λimm434;* Δ*his1::hisG /* Δ*his1::hisG;* Δ*arg4::hisG /* Δ*arg4::hisG*; *RPS10 /* Δ*rps10*::pCIP30 | [4] |
| *als3*Δ | BWP17 | homozygous mutant | [5] |
|  |  | Δ*ura3::* λ*imm434 ::URA3-IRO1 /* Δ*ura3::* λ*imm434;* Δ*als3 ::ARG4 /* Δ*als3 ::HIS1* |  |
| *als3*Δ::ALS3 | BWP17 | complemented mutant | [5] |
|  |  | Δ*ura3::* λ*imm434 ::URA3-IRO1 /* Δ*ura3::* λ*imm434;* Δ*als3 ::ARG4::ALS3* / Δ*als3 ::HIS1* |  |
| *bcr1*Δ | BWP17 | homozygous mutant | [6] |
|  |  | Δ*his1 ::hisG::pHIS1 / his1::hisG;* Δ*bcr1 ::ARG1 /* Δ*bcr1 ::URA3* |  |
| *bcr1*Δ::BCR1 | BWP17 | complemented mutant | [6] |
|  |  | Δ*bcr1::Tn7-UAU1::pHIS1-TEF1-BCR1 /* Δ*bcr1::Tn7-URA3* |  |
| *cka2*Δ | BWP17 | homozygous mutant | [7] |
|  |  | Δ*ura3::* λ*imm434 ::URA3-IRO1 /*Δ*ura3::* λ*imm434;* Δ*cka2::HIS1 /* Δ*cka2::ARG4* |  |
| *cph1*Δ | CAI4 | homozygous mutant | [8] |
|  |  | Δ*cph1::hisG / cph 1:: hisG-URA-hisG* |  |
| *dfg16*Δ | CAI4 | homozygous mutant | [9] |
|  |  | Δ*dfg16::hisG /* Δ*dfg16::hisG*; *RPS10 /* Δ*rps10*::pCIP10 |  |
| *dfg16*Δ::DFG16 | CAI4 | complemented mutant | [9] |
|  |  | Δ*dfg16::hisG /* Δ*dfg16::hisG*; *RPS10 /* Δ*rps10*::pCIP30-*DFG16* |  |
| *eed1*Δ | BWP17 | homozygous mutant | [10] |
|  |  | Δ*ipf946::HIS1*/ Δ*ipf946::ARG4; RPS10 /* Δ*rps10*::pCIP30 |  |
| *eed1*Δ::EED1 | BWP17 | complemented mutant | [10] |
|  |  | EED1/EED1; *RPS10 /* Δ*rps10*::pCIP30 |  |
| *efg1*Δ | CAI4 | homozygous mutant | [11] |
|  |  | Δ*efg1*::*hisG* / Δ*efg1*::*hisG-URA3-hisG* |  |
| *efg1*Δ::EFG1 | CAI4 | complemented mutant | [4] |
|  |  | Δ*efg1*::*hisG-EFG1* / Δ*efg1*::*hisG-URA3-hisG* |  |
| *efg1*Δ*cph1*Δ | CAI4 | homozygous double mutant | [11] |
|  |  | Δ*cph1*::*hisG* / *cph1*::*hisG*; Δ*efg1*::*hisG* / Δ*efg1*::*hisG-URA3-hisG* |  |
| *mnt1*Δ | CAI4 | homozygous mutant | [12] |
|  |  | Δ*mnt1*::*hisG-URA3-hisG* / Δ*mnt1*::*hisG* |  |
| *mnt1*Δ::MNT1 | CAI4 | complemented mutant | [12] |
|  |  | Δ*mnt1*::*hisG-URA3-hisG*/Δ*mnt1*::*hisG; RPS10 / Δrps10*::pCIP10-*MNT1* |  |
| *ras1*Δ | CAI4 | homozygous mutant | [13] |
|  |  | Δ*ras1D::hisG /* Δ*ras1D::hph-URA3-hph* |  |
| *rim101*Δ | CAI4 | homozygous mutant | [14] |
|  |  | Δ*prr2::hisG /* Δ*prr2::hisG-URA3-hisG* |  |
| *sap1-3*Δ | CAI4 | homozygous triple mutant | [15] |
|  |  | Δ*sap1*::*hisG* / Δs*ap1*::*hisG;* Δ*sap2*::*hisG* / Δ*sap2*::*hisG;* Δ*sap3*::*hisG* / Δ*sap3*::*hisG-URA3-hisG* |  |

| **Strain** | **Background** | **Genotype or description^a^** | **Source or reference** |
| --- | --- | --- | --- |
| *sap4-6*Δ | CAI4 | homozygous triple mutant | [16] |
|  |  | Δ*sap6*::*hisG* / Δs*ap1*::*hisG;* Δ*sap4*::*hisG* / Δ*sap4*::*hisG;* Δ*sap5*::*hisG* / Δ*sap5*::*hisG-URA3-hisG* |  |
| *tec1*Δ | BWP17 | homozygous mutant | [17] |
|  |  | Δ*his1 ::hisG::pHIS1 /*Δ *his1::hisG;* Δ*tec1::Tn7-UAU1 /*Δ *tec1::Tn7-URA3* |  |
| *tpk2*Δ | CAI4 | homozygous mutant | [18] |
|  |  | Δ*tpk2::hisG / tpk2::hisG-URA-hisG* |  |

^a^Genotype: differences to parental strain

**References**

1. Gillum AM, Tsay EY, Kirsch DR (1984) Isolation of the *Candida albicans* gene for orotidine-5'-phosphate decarboxylase by complementation of *S. cerevisiae* ura3 and *E. coli* pyrF mutations. Mol Gen Genet 198: 179-182.

2. Fradin C, De Groot P, MacCallum D, Schaller M, Klis F, et al. (2005) Granulocytes govern the transcriptional response, morphology and proliferation of *Candida albicans* in human blood. Mol Microbiol 56: 397-415.

3. Murad AM, Lee PR, Broadbent ID, Barelle CJ, Brown AJ (2000) CIp10, an efficient and convenient integrating vector for *Candida albicans*. Yeast 16: 325-327.

4. Wilson RB, Davis D, Mitchell AP (1999) Rapid hypothesis testing with *Candida albicans* through gene disruption with short homology regions. J Bacteriol 181: 1868-1874.

5. Phan QT, Myers CL, Fu Y, Sheppard DC, Yeaman MR, et al. (2007) Als3 Is a *Candida albicans* Invasin That Binds to Cadherins and Induces Endocytosis by Host Cells. PLoS Biol 5: e64.

6. Nobile CJ, Andes DR, Nett JE, Smith FJ, Yue F, et al. (2006) Critical role of Bcr1-dependent adhesins in *C. albicans* biofilm formation in vitro and in vivo. PLoS Pathog 2: e63.

7. Chiang LY, Sheppard DC, Bruno VM, Mitchell AP, Edwards JE, Jr., et al. (2007) *Candida albicans* protein kinase *CK2* governs virulence during oropharyngeal candidiasis. Cell Microbiol 9: 233-245.

8. Liu H, Kohler J, Fink GR (1994) Suppression of hyphal formation in *Candida albicans* by mutation of a STE12 homolog. Science 266: 1723-1726.

9. Thewes S, Kretschmar M, Park H, Schaller M, Filler SG, et al. (2007) *In vivo* and *ex vivo* comparative transcriptional profiling of invasive and non-invasive *Candida albicans* isolates identifies genes associated with tissue invasion. Mol Microbiol 63: 1606-1628.

10. Zakikhany K, Naglik JR, Schmidt-Westhausen A, Holland G, Schaller M, et al. (2007) *In vivo* transcript profiling of *Candida albicans* identifies a gene essential for interepithelial dissemination. Cell Microbiol 9: 2938-2954.

11. Lo HJ, Kohler JR, DiDomenico B, Loebenberg D, Cacciapuoti A, et al. (1997) Nonfilamentous *C. albicans* mutants are avirulent. Cell 90: 939-949.

12. Buurman ET, Westwater C, Hube B, Brown AJ, Odds FC, et al. (1998) Molecular analysis of CaMnt1p, a mannosyl transferase important for adhesion and virulence of *Candida albicans*. Proc Natl Acad Sci U S A 95: 7670-7675.

13. Feng Q, Summers E, Guo B, Fink G (1999) Ras signaling is required for serum-induced hyphal differentiation in *Candida albicans*. J Bacteriol 181: 6339-6346.

14. Ramon AM, Fonzi WA (2003) Diverged binding specificity of Rim101p, the *Candida albicans* ortholog of PacC. Eukaryot Cell 2: 718-728.

15. Kretschmar M, Felk A, Staib P, Schaller M, Hess D, et al. (2002) Individual acid aspartic proteinases (Saps) 1-6 of *Candida albicans* are not essential for invasion and colonization of the gastrointestinal tract in mice. Microb Pathog 32: 61-70.

16. Sanglard D, Hube B, Monod M, Odds FC, Gow NA (1997) A triple deletion of the secreted aspartyl proteinase genes SAP4, SAP5, and SAP6 of Candida albicans causes attenuated virulence. Infect Immun 65: 3539-3546.

17. Schweizer A, Rupp S, Taylor BN, Rollinghoff M, Schroppel K (2000) The TEA/ATTS transcription factor CaTec1p regulates hyphal development and virulence in *Candida albicans*. Mol Microbiol 38: 435-445.

18. Sonneborn A, Bockmuhl DP, Gerads M, Kurpanek K, Sanglard D, et al. (2000) Protein kinase A encoded by TPK2 regulates dimorphism of *Candida albicans*. Mol Microbiol 35: 386-396.
